# A summary-statistics-based approach to examine the role of serotonin transporter promoter tandem repeat polymorphism in psychiatric phenotypes

**DOI:** 10.1038/s41431-021-00996-6

**Published:** 2021-12-23

**Authors:** Arunabha Majumdar, Preksha Patel, Bogdan Pasaniuc, Roel A. Ophoff

**Affiliations:** 1 Department of Mathematics, Indian Institute of Technology Hyderabad, Kandi, Telangana India; 2 Department of Pathology and Laboratory Medicine, David Geffen School of Medicine, University of California, Los Angeles, CA USA; 3 Center for Neurobehavioral Genetics, Semel Institute for Neuroscience and Human Behavior, University of California, Los Angeles, CA USA; 4 Department of Computational Medicine, David Geffen School of Medicine, University of California, Los Angeles, CA USA

**Keywords:** Behavioural genetics, Genetic association study

## Abstract

In genetic studies of psychiatric disorders in the pre-genome-wide association study (GWAS) era, one of the most commonly studied loci is the serotonin transporter (SLC6A4) promoter polymorphism, a 43-base-pair insertion/deletion polymorphism in the promoter region (5-HTTLPR). The genetic association signals between 5-HTTLPR and psychiatric phenotypes, however, have been inconsistent across many studies. Since the polymorphism cannot be tested via available SNP arrays, we had previously proposed an efficient machine learning algorithm to predict the genotypes of 5-HTTLPR based on the genotypes of eight nearby SNPs, which requires access to individual-level genotype and phenotype data. To utilize the advantage of publicly available GWAS summary statistics obtained from studies with very large sample sizes, we develop a GWAS summary-statistics-based approach for testing the variable number of tandem repeat (VNTR) associations with various phenotypes. We first cross-verify the accuracy of the summary-statistics-based approach for 61 phenotypes in the UK Biobank. Since we observed a strong similarity between the predicted individual-level 5-HTTLPR genotype-based approach and the summary-statistics-based approach, we applied our method to the available neurobehavioral GWAS summary statistics data obtained from large-scale GWAS. We found no genome-wide significant evidence for association between 5-HTTLPR and any of the neurobehavioral traits. We did observe, however, genome-wide significant evidence for association between this locus and human adult height, BMI, and total cholesterol. Our summary-statistics-based approach provides a systematic way to examine the role of VNTRs and related types of genetic polymorphisms in disease risk and trait susceptibility of phenotypes for which large-scale GWAS summary statistics data are available.

## Introduction

Large-scale human genetic studies have resulted in major breakthroughs for our understanding of the genetic architecture of complex human traits. SNP arrays for genome-wide association studies (GWAS) in up to millions of subjects are extensively used for the detection of common risk alleles for polygenic disorders such as schizophrenia [1] and bipolar disorder [2]. On the other hand, the approach of high-throughput sequencing in tens of thousands of individuals has identified rare and deleterious sequence variants contributing to neurodevelopmental disorders such as autism spectrum disorder [3] and schizophrenia [4]. One type of genetic variation that was extensively studied before the advances in genomic technology, the variable number of tandem repeats (VNTRs) class of variants [5], has eluded further large-scale analysis in the genomic era. SNP arrays allow for the high-throughput collection of single nucleotide variants but not for VNTRs, microsatellite polymorphisms, and related genetic variants that are also abundant in the human genome.

In genetic studies of psychiatric disorders, one of the most commonly studied locus in the pre-GWAS era [6], is the serotonin transporter (5-HTT or SLC6A4) promoter polymorphism, a 43-base-pair insertion/deletion polymorphism in the promoter region (5-HTTLPR) with a long (L) and a short (S) allele. The SLC6A4 gene was long considered a strong candidate gene for neurobehavioral traits and disorders. The reason is that many antidepressant drugs were reported to selectively inhibit the function of the serotonin transporter (5-HTT or SLC6A4), the gene product of which mediates the reuptake of monoamine serotonin (5-HT), a key neurotransmitter in the brain. Consequently, genetic variants such as the 5-HTTLPR were considered important factors to modulate the expression of the serotonin transporter protein under certain physiologic conditions. However, the genetic association signal findings between 5-HTTLPR and psychiatric and neurobehavioral phenotypes have been found to be inconsistent across studies, few indicating an association, for example, when interacting with stressful life events [7], but a meta-analysis with a total sample size of more than 14,000 participants failed to provide any evidence for association with depression with or without stressful life events [8]. Another systematic gene x environment interaction (GxE) meta-analysis of this locus showed that the published studies are underpowered and that the main effects of 5-HTTLPR genotype and the interaction effect of stressful life events on risk of depression are negligible [9]. Another meta-analysis of 54 studies that included 40,749 subjects, in which environmental effects as well as phenotype outcomes were taken very broadly, reported a positive GxE effect with the S allele associated with increased stress sensitivity [10].

Since the 5-HTTLPR VNTR cannot be tested via available SNP arrays, we previously proposed an efficient machine learning algorithm to predict the genotypes of 5-HTTLPR based on genotypes of eight nearby SNPs [11]. The predicted VNTR genotypes can then be used for association testing with a phenotype of interest. However, this approach requires access to individual-level genotype data for the eight SNPs and phenotype data; hence, it cannot utilize the advantage of publicly available GWAS summary statistics obtained from studies with very large sample sizes across various neurobehavioral phenotypes. In order to revisit the 5-HTTLPR for genetic analysis in the largest study samples available, we developed and applied a GWAS summary-statistics-based approach for association testing of this locus.

In short, in a training dataset that has PCR-based genotypes for 5-HTTLPR and the eight SNPs considered for our machine learning algorithm [11], we fit a linear model to estimate the allele dosage of 5-HTTLPR based on the eight SNPs. We use this prediction model to impute the allele dosage of 5-HTTLPR in a GWAS dataset (where 5-HTTLPR genotypes are not available) and perform association testing with the phenotype. We devise a summary-statistics-based version of this two-step procedure in which we only need the GWAS summary statistics at the eight SNPs for the phenotype. We first cross-verify the accuracy of the summary-statistics-based approach for 61 phenotypes in UK Biobank (UKBB). We contrast the association statistics computed by the summary-statistics-based approach and our previous genotype-based method. Since we observed a strong similarity between the two approaches, we applied our method to the available neurobehavioral GWAS summary statistics data with large sample size as well as of other disorders and traits. We found no genome-wide significant evidence for association between the 5-HTTLPR and any of the neurobehavioral traits. We did observe, however, genome-wide significant evidence for association between this locus and human adult height, body mass index (BMI), and total cholesterol. Our approach provides a systematic way to re-assess and examine the role of specific VNTRs and related types of genetic polymorphisms in disease risk and trait susceptibility of phenotypes for which summary-level data from large-scale GWAS are available.

## Methods

### Prediction of 5-HTTLPR genotypes based on tag SNPs

In an ideal scenario, if the genotype data of 5-HTTLPR is available along with the phenotype of interest in a sufficiently large study sample, we can directly assess the association between 5-HTTLPR and the phenotype. The PCR-based assay to genotype the serotonin transporter promoter polymorphism has suffered from technical difficulties as demonstrated by the lack of Hardy–Weinberg equilibrium in genotype data for this locus [9]. With the rise of use of SNP genotyping arrays, we previously proposed a method to predict the 5-HTTLPR VNTR genotypes based on nearby tag SNPs, which also requires the individual-level genotype data of the tag SNPs. We now move one step further, beyond the need to access individual-level genotype data but to leverage the largest available sample sizes for genetic studies used for GWAS. Our approach to examine the 5-HTTLPR VNTR is similar to the summary-statistics-based method proposed by Gusev et al. [12] to evaluate the association between the genetic component of a gene’s expression and a phenotype.

The serotonin transporter (SLC6A4) promoter polymorphism is a tandem repeat polymorphism, also called the 5-HTTLPR, with two alleles: one long allele (L) and one short allele (S), hence the three possible genotypes SS, SL, and LL. Using additive coding, i.e., counting the number of L alleles in a genotype, the coded genotype can take three possible values: 0, 1, 2. Suppose, we have a reference dataset containing the genotype data of 5-HTTLPR and the surrounding eight SNPs tagging 5-HTTLPR (Table 1), which we considered in our previous work [11]. We use an individual-level genotype dataset for these nine variants (5-HTTLPR and eight tag SNPs) that consists of 276 Dutch individuals comprising 126 normal individuals and 150 individuals diagnosed with schizophrenia [11]. We consider a linear regression model to estimate the component of the 5-HTTLPR genotypes that can be predicted by the eight tag SNPs.

1 $${{g}} = \delta + \gamma _1x_1 + \gamma _2{{{{{{{\mathrm{x}}}}}}}}_2 + \,\cdots\, + \gamma _8{{{{{{{\mathrm{x}}}}}}}}_8 + {\it{ \in }}$$

Here *g* denotes the genotype of 5-HTTLPR, and *x*_*j*_ denotes the genotype of *j*th tag SNP, *j* = 1,…,8. Let *γ*_*j*_ denote the regression coefficient corresponding to *j*th tag SNP’s genotype. We consider the genotype vector of each tag SNP to be normalized (zero mean and unity variance). We note that the predicted component of 5-HTTLPR genotypes, $\hat g$, obtained from the linear regression can be continuous. The least square estimation of *γ*_1_,…,*γ*_8_ does not depend on the distribution of the noise *ϵ*. Since the sample size of the Dutch dataset (*n* = 276) is much larger than the number of tag SNPs, a linear regression model is adequate for model fitting instead of a penalized regression.

Let *X* denote the genotype data matrix for the eight tag SNPs (Table 1). Using the multiple linear regression model stated above, we obtain the least square estimate (LSE) of *γ* = (*γ*_1_,…,*γ*_8_)’ as: $\hat \gamma = \left( {X^{\prime}X} \right)^{ - 1}X^{\prime}G$, where *G* is the genotype vector for 5-HTTLPR. Once we estimate *γ*, it is considered fixed in the down-stream analysis. If the 5-HTTLPR genotypes are missing in a different dataset, but the genotypes of the eight tag SNPs are available, we can use $\widehat G = \widehat \gamma x$ to predict the 5-HTTLPR genotypes, where *x* is the genotype vector for the tag SNPs.

**Table 1:** Tag SNPs of 5-HTTLPR.

Chromosome	Base-pair position (GRCh38)	SNP	LD
17	30245735	rs1487971	0.54
17	30263512	rs2129785	0.37
17	30262460	rs11651241	0.25
17	30421334	rs4794873	0.1
17	30365556	rs887469	–0.02
17	30787445	rs1061342	0.04
17	30161773	rs4494608	–0.07
17	29448649	rs7217677	0.17

The fourth column provides the linkage disequilibrium (LD) between 5-HTTLPR and its tag SNPs.

### Evaluating association between 5-HTTLPR and a phenotype

Suppose, for *n* unrelated individuals in a new dataset, *Y* denotes the phenotype of interest, *G* denotes the genotype of 5-HTTLPR that is unobserved. We assume that the individual-level data for *Y* and genotypes of the tag SNPs are available, but the 5-HTTLPR genotypes are missing. Consider a linear regression of *Y* on unobserved *G* as follows:

2 $$Y = G\alpha + e$$

Let *X* denote the genotype data matrix for the eight tag SNPs. Since *G* is unobserved, we can use our previously proposed machine learning method [11] to predict *G* based on *X*, and perform a test for association between predicted *G* and *Y*.

When individual-level data of *Y* and genotype data of the tag SNPs are also not available, we propose the following summary-statistics-based approach. We use the prediction model that was obtained from linear regression fitted in the Dutch reference dataset (Eq. 1) to predict the genotypes of 5-HTTLPR as: $\hat G = X\hat \gamma$. Thus, we consider $Y = \hat G\alpha + e$, where *e* is the random error. In this paper, we assume that $\widehat \gamma$ is the same between the Dutch and other European populations. Next, we obtain the LSE of *α* in the linear regression $Y = \hat G\alpha + e$.

3 $$\hat \alpha = \left( {\hat G^{\prime}\hat G} \right)^{ - 1}\hat G^{\prime}Y = \frac{1}{{n\left( {\hat \gamma ^{\prime}V\hat \gamma } \right)}}\hat \gamma^{\prime}X^{\prime}Y$$

Here, *V* is the LD matrix for the eight tag SNPs. We derive the above formula using the facts that, $\hat G = X\hat \gamma$ and *X’X* = *nV*, since the genotype vector for each tag SNP is normalized. Suppose, *Y* is also separately regressed on the genotype of *j*th tag SNP as follows:

4 $$Y = X_j\beta _j + {\it{ \in }}_j$$

where *Y* and *X*_*j*_ are normalized. Let *β*_*j*_ denote the marginal GWAS effect size of *j*th tag SNP on *Y*. LSE of *β*_*j*_ is given by: $\hat \beta _j = ( {X^{\prime}_jX_j} )^{ - 1}X^{\prime}Y = \frac{1}{n}X^{\prime}Y \Rightarrow X^{\prime}Y = n\hat \beta _j$. Since *X*_*j*_ is normalized, $X^{\prime}_jX_j = n$. Denote the vector of estimated marginal GWAS effect size across the eight tag SNPs as: $\hat \beta = ( {\hat \beta _1, \ldots ,\hat \beta _8} )^\prime$. Hence, for the tag SNPs considered together: $X^{\prime}Y = n\hat \beta$. Thus, we can rewrite the expression of LSE of the effect size of 5-HTTLPR on *Y*, $\hat \alpha$ (Eq. 3), as follows:

5 $$\hat \alpha = \frac{1}{{n\left( {\hat \gamma ^{\prime}V\hat \gamma } \right)}}\hat \gamma ^{\prime}\left( {n\hat \beta } \right) = \frac{{\hat \gamma^{\prime}\hat \beta }}{{\hat \gamma^{\prime}V\hat \gamma }}$$

Next, we derive the expression of standard error of $\hat \alpha$. Let us define the constant $K_{wv} = \hat \gamma^{\prime}V\hat \gamma$. Hence,

6 $${\mathop{{{{{{{\rm{var}}}}}}}}} \left( {\hat \alpha } \right) = \frac{1}{{K_{wv}^2}}{\mathop{{{{{{{\rm{var}}}}}}}}} \left( {\hat \gamma^{\prime}\hat \beta } \right) = \frac{{\hat \gamma^{\prime}{\mathop{{{{{{{\rm{cov}}}}}}}}} \left( {\hat \beta } \right)\hat \gamma }}{{K_{wv}^2}}$$

Here, ${\mathop{{{{{{{\rm{cov}}}}}}}}} ( {\hat \beta } ) = \frac{{\sigma _y^2}}{n}V$ is the variance-covariance matrix of $\hat \beta$, where $\sigma _y^2 = {\mathop{{{{{{{\rm{var}}}}}}}}} \left( Y \right)$ and *V* is the LD matrix of the tag SNPs. So,

7 $${\mathop{{{{{{{\rm{var}}}}}}}}} \left( {\hat \alpha } \right) = \frac{1}{{K_{wv}^2}}\left[ {\hat \gamma ^{\prime}{\mathop{{{{{{{\rm{cov}}}}}}}}} \left( {\hat \beta } \right)\hat \gamma } \right] = \frac{1}{{K_{wv}^2}}\left[ {\frac{{\sigma _y^2}}{n}\hat \gamma ^{\prime}V\hat \gamma } \right] = \frac{1}{{K_{wv}^2}}\left[ {\frac{{\sigma _y^2}}{n}K_{wv}} \right] = \frac{{\sigma _y^2}}{{nK_{wv}}}$$

In practice, *Y* is normalized before running a GWAS. Hence, we assume that $\sigma _y^2 = 1$. Suppose *Z*_*β*_ be the vector of marginal *z*-scores of associations between *Y* and the tag SNPs. We can rewrite the expression of *Z*-statistics for testing *α* = 0 as follows:

8 $$Z_\alpha = \frac{{\hat \alpha }}{{\surd {\mathop{{{{{{{\rm{var}}}}}}}}} \left( {\hat \alpha } \right)}} = \frac{{\hat \gamma ^{\prime}Z_\beta }}{{\surd \left( {\hat \gamma ^{\prime}V\hat \gamma } \right)}}$$

For a sufficiently large sample size, each element of *Z*_*β*_ follows a normal distribution. Hence, *Z*_*α*_ also follows a normal distribution asymptotically. If *Z*_*α*_ = *z*, a *p* value of association between 5-HTTLPR and *Y* can be obtained as *P*(*U* > *z*^2^), where *U* follows a *χ*^2^ distribution with one degree of freedom.

Thus, given $Z_\beta ,\gamma ,V$, we can test *α* = 0. Therefore, using summary-level data on genetic association between a phenotype *Y* and the tag SNPs of 5-HTTLPR, the estimated prediction model for 5-HTTLPR genotypes (based on the Dutch reference dataset), and an estimate of the LD matrix of the tag SNPs, we can test for association between 5-HTTLPR and *Y*. We do not require any individual-level phenotype data and genotype data of 5-HTTLPR and related tag SNPs. We can use an estimate of *V*, the LD matrix of the tag SNPs, obtained based on the European individuals in 1000 genome data. To further increase the number of individuals in the LD reference panel, we combined the 276 Dutch individuals in our reference dataset with the European individuals in 1000 genome. We also provide the LD between 5-HTTLPR and its tag SNPs in Table 1. In the rest of this paper, we refer to our summary-statistics-based approach as VNTR.s.

## Validation of summary-statistics-based approach in UK Biobank

In order to cross-verify the accuracy of the summary-statistics-based approach (VNTR.s), we conducted a phenome-wide association study for 5-HTTLPR and 61 phenotypes in the UKBB based on both of the individual-level prediction of 5-HTTLPR genotypes [11] and the summary-statistics-based approach VNTR.s. We contrast the results of association analyses obtained by these two methods to assess the accuracy of VNTR.s. We discuss the procedure of analyzing a single phenotype in UKBB (e.g., height).

First, we implement our previous machine learning approach (vertex discriminant analysis [11]) for 287,969 white British individuals in UKBB. We used the individual-level genotype data of the tag SNPs (Table 1) to predict 5-HTTLPR genotypes for the UKBB individuals. In our previous work [11], we estimated the coefficients of the 5-HTTLPR genotype prediction model based on the Finn dataset in which individual-level genotype data for both 5-HTTLPR and the tag SNPs were available for 2147 normal participants [11]. We used the same coefficients (Supplementary Table S3) to predict the 5-HTTLPR genotypes in UKBB. Next, we perform a linear regression of the phenotype on the predicted 5-HTTLPR genotypes to compute a *p* value of association while adjusting for relevant covariates, such as, age, sex, and top 20 principal components (PCs) of genetic ancestry. We refer to this approach as VNTR.g.

Next, we apply VNTR.s for the same phenotype in UKBB. We perform a linear regression of the phenotype on the genotype of each tag SNP of 5-HTTLPR, while adjusting for the same set of covariates, age, sex, and top 20 genetic ancestry of PCs. We obtain the summary statistics of association between the phenotype and each tag SNP from these regressions. We now assume that only the summary statistics for each tag SNP are available. Using the summary statistics and estimate of the LD matrix for the tag SNPs (as described above), we apply VNTR.s to compute the *p* value of association between 5-HTTLPR and the phenotype.

We applied VNTR.g and VNTR.s for 61 phenotypes in UKBB to compute the *p* values of association along with *z*-scores. We note that the genotype data for one of the tag SNPs, rs1061342 on chromosome 17 (Table 1), was missing from the UKBB individual-level genotype dataset available to us. Albeit under this limitation, we implemented VNTR.g and VNTR.s based on the remaining seven tag SNPs (Table 1). In our previous work [11], we evaluated the relative contribution of the tag SNPs to the prediction accuracy of the 5-HTTLPR genotypes with respect to partial and cumulative *R*^2^. The partial *R*^2^ for rs1061342, the missing SNP in UKBB, was very modest (Figure 1 in Lu et al. [11]) and the cumulative *R*^2^ increased from 0.83 to 0.84 after adding rs1061342 to the prediction model (sixth row of Table 2 in Lu et al. [11]). The total cumulative *R*^2^ of the full prediction model was 0.85. Thus, from the viewpoint of a stepwise regression, the prediction accuracy gets saturated before rs1061342 is added to the prediction model. Therefore, we emphasize that the comparison between VNTR.g and VNTR.s remains meaningful, albeit under the limitation of our UKBB individual-level genotype dataset.

Next, we contrast the results obtained by VNTR.g and VNTR.s for 61 UKBB phenotypes. Encouragingly, the *p* values obtained by the two approaches are very similar (Table 2). For example, for FEV1–FVC ratio, VNTR.g produced a *p* value of 1.6 × 10^–8^ and VNTR.s produced a *p* value of 3.2 × 10^–8^ (Table 2). For Albumin, VNTR.g produced a *p* value of 0.95 and VNTR.s produced a *p* value of 0.93 (Table 2). In the following, we provide an overall summary of the similarity.

**Table 2:** Association *p* values for 61 phenotypes in UK Biobank obtained by VNTR.g and VNTR.s.

Phenotype	VNTR.g	VNTR.s	Phenotype	VNTR.g	VNTR.s
Height	6.01E–12	3.95E–15	Sodium in urine	0.17	0.16
FEV1–FVC ratio	1.56E–08	3.15E–08	Gamma glutamyltransferase	0.17	0.09
Mean platelet volume	7.26E–07	3.12E–08	FVC	0.18	0.08
Platelet distribution width	1.87E–05	1.75E–05	Cystatin-C	0.22	0.23
Testosterone	0.0002	0.0005	Calcium	0.24	0.15
Alcohol intake frequency	0.0002	0.0001	RBC distribution width	0.27	0.57
Basal metabolic rate	0.0005	0.0001	Alkaline phosphatase	0.28	0.49
SHBG	0.001	0.002	Corneal hysteresis	0.3	0.28
Atherosclerosis-related	0.001	0.001	Lymphocyte count	0.31	0.24
Platelet count	0.003	0.002	Potassium in urine	0.34	0.26
Mean corpuscular hemoglobin	0.02	0.2	Body mass index	0.34	0.3
Overall health rating	0.02	0.01	Creatinine in urine	0.37	0.47
IGF-1	0.03	0.03	Apolipoprotein B	0.4	0.4
Lipoprotein-A	0.03	0.05	Apolipoprotein A	0.41	0.58
White blood cell count	0.04	0.05	Direct bilirubin	0.41	0.58
Urate	0.05	0.03	Triglycerides	0.43	0.48
Hemoglobin A1c	0.05	0.01	High light scatter reticulocyte count	0.48	0.22
Aspartate aminotransferase	0.06	0.11	LDL direct	0.49	0.53
Eosinophil count	0.09	0.2	Phosphate	0.53	0.43
Waist–hip ratio	0.1	0.11	C-reactive protein	0.55	0.68
Alanine aminotransferase	0.11	0.1	Microalbumin in urine	0.6	0.38
Asthma	0.11	0.12	Monocyte count	0.61	0.42
Mean sphered cell volume	0.13	0.66	Autoimmune-related	0.64	0.56
Creatinine	0.13	0.34	Cholesterol	0.64	0.71
Tanning (quantitative)	0.13	0.1	Glucose	0.69	0.93
BMD Heel T-score	0.14	0.14	Hair color	0.8	0.64
Total protein	0.16	0.13	HDL cholesterol	0.91	0.99
Total bilirubin	0.16	0.33	Oestradiol higher than 212 pmol/L	0.91	0.82
RBC count	0.16	0.58	Urea	0.92	0.79
Sodium	0.17	0.16	Albumin	0.95	0.93
			Rheumatoid factor higher than 16 U/mL	0.99	0.92

**Fig. 1:**
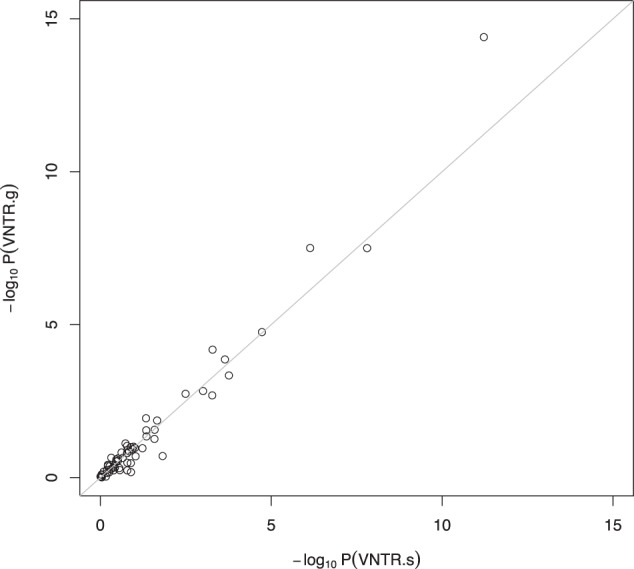
Plot of –log_10_(*p* value) obtained by VNTR.g and VNTR.s for 61 phenotypes in the UK Biobank. If *y* denotes –log_10_(*p* value) for VNTR.g and *x* denotes –log_10_(*p* value) for VNTR.s, the estimated linear regression equations were obtained as: *y* = –0.16 + 1.15*x* and *x* = 0.19 + 0.83*y*.

For a UKBB phenotype, let P.g and P.s denote the *p* values obtained by VNTR.g and VNTR.s, respectively. Let *y* and *x* denote –log_10_(P.g) and –log_10_(P.s), respectively. Pearson’s correlation coefficient between *y* and *x* based on 61 UKBB phenotypes was estimated as 0.98. Spearman’s rank correlation coefficient was estimated to be 0.9. If we fit a linear regression of *y* on *x*, we obtain the following regression equation: *y* = –0.16 + 1.15*x*. Here, the slope regression coefficient was estimated as 1.15 with a standard error of 0.03, and the intercept coefficient was estimated to be –0.16 with a standard error of 0.07. Similarly, we regressed *x* on *y* and obtained the following regression equation: *x* = 0.19 + 0.83*y*. We also performed the non-parametric Kolmogorov–Smirnov (KS) test to evaluate if *x* and *y* have the same distribution. The KS test *p* value was 0.99 that strongly implies that *x* and *y* have the same underlying distribution. A plot of the observed values of *x* and *y* (Fig. 1) displays good concordance between the results obtained by VNTR.g and VNTR.s.

Thus, the overall analysis of 61 UKBB phenotypes demonstrates an excellent agreement between the outputs of VNTR.g and VNTR.s. This validates the use of VNTR.s for testing the association between 5-HTTLPR and a phenotype of interest based on publicly available summary-level data for association between the tag SNPs and the phenotype.

## Application of the summary-statistics-based approach to various phenotypes

Using publicly available summary statistics data from GWAS with large sample sizes, we implemented VNTR.s to study association of 5-HTTLPR with a range of phenotypes mainly focusing on psychiatric and neurobehavioral phenotypes. We applied VNTR.s to nine psychiatric phenotypes in the psychiatric genetics consortium (PGC). We then examined 131 phenotypes using summary statistics available at the GWAS Atlas [13] (https://atlas.ctglab.nl/) from psychiatric, cognitive, neurological phenotype domains, each based on reasonably powered GWAS with sample size of *N* > 50,000 subjects. Most of these studies are based on UKBB data, and all the studies are based on European ancestry. Since individual-level genotype and phenotype data used in these GWAS are not publicly available, it is very challenging to implement VNTR.g in this context.

Based on a genome-wide significant threshold of *p* value 5 × 10^–8^, we did not find any significant association between 5-HTTLPR and the psychiatric phenotypes in PGC (Table 3). For example, the smallest *p* value was 0.002 for bipolar disorder (Table 3). Among the psychiatric phenotypes analyzed from GWAS atlas, we also did not find any significant association (Table 4 and Supplementary Tables S1 and S2). We provide the GWAS marginal association *p* values for each pair of the tag SNPs and the psychiatric phenotypes in PGC (Supplementary Table S4).

**Table 3:** 5-HTTLPR association *p* values obtained by VNTR.s for the psychiatric phenotypes in the psychiatric genetics consortium.

Disorder	Number of cases	Number of controls	*p* value
Bipolar disorder	20,352	31,358	0.002
Attention deficit hyperactivity disorder	19,099	34,194	0.01
Eating disorder	16,992	55,525	0.01
Post-traumatic stress disorder	30,000	170,000	0.1
Schizophrenia	13,833	18,310	0.2
Autism spectrum disorder	18,381	27,969	0.3
Major depressive disorder	59,851	113,154	0.4
Tourette syndrome	4819	9488	0.6
Alcohol dependence	14,904	37,944	0.8

**Table 4:** 5-HTTLPR association *p* values obtained by VNTR.s for the first set of psychiatric phenotypes in GWAS atlas (https://atlas.ctglab.nl/).

Phenotype	*p* value
Alcohol intake frequency	1.73E–06
Ever vs never smokers	0.0002
Champagne/white wine intake	0.003
Able to pay rent/mortgage as an adult	0.009
Subjective well being	0.009
Extraversion	0.009
Ever vs never drinkers	0.02
Frequency of inability to cease drinking in the last year	0.02
Frequency of consuming six or more units of alcohol	0.02
Frequency of feeling guilt or remorse after drinking alcohol in the last year	0.04
Major depressive disorder, single episode	0.06
Recent trouble relaxing	0.06
Former vs current drinkers	0.08
Current tobacco smoking	0.08
Weight change during worst episode of depression	0.1
Light smokers	0.1
Former vs current smokers	0.11
Depression possibly related to stressful or traumatic event	0.12
Ever had period extreme irritability	0.12
Recent worrying too much about different things	0.13
Drinks per week	0.17
Trouble falling or staying asleep, or sleeping too much	0.2
Frequency of memory loss due to drinking alcohol in the last year	0.2
Ever depressed for a whole week	0.2
Reason for reducing amount of alcohol drunk: health precaution	0.23
Thoughts of death during worst depression	0.24
Felt loved as child	0.25
Frequency of unenthusiasm/disinterest in last 2 weeks	0.26
Frequency of tiredness/lethargy in last 2 weeks	0.28
Someone to take to doctor when needed as a child	0.28
Fraction of day affected during worst episode of depression	0.29
Frequency of depressed days during worst episode of depression	0.29
Been in serious accident believed to be life-threatening	0.29
Ever worried more than most people would in similar situation	0.29
Recent restlessness	0.3
Why stopped smoking: financial reasons	0.3
Been in a confiding relationship as an adult	0.31
Ever had prolonged feelings of sadness or depression	0.31

**Fig. 2:**
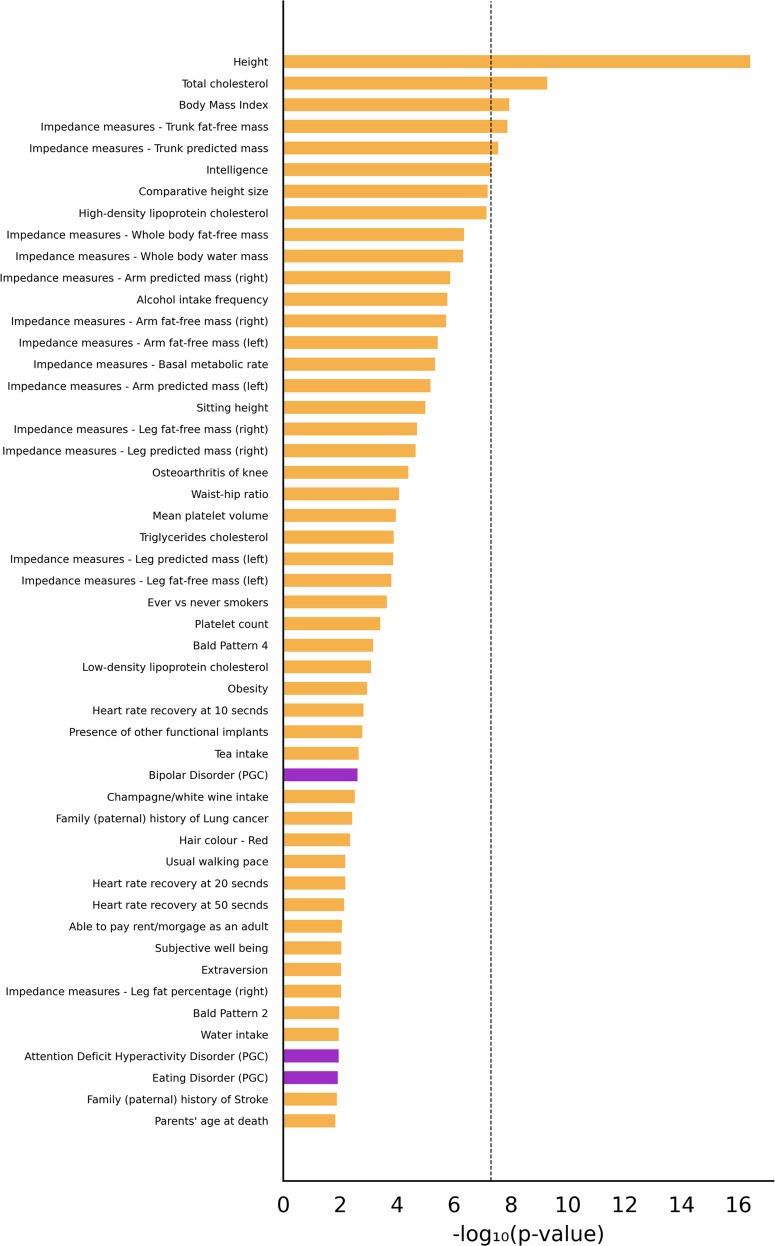
Plot of –log_10_(*p* values) for 50 phenotypes with most significant *p* values obtained by VNTR.s. Horizontal bars for the psychiatric phenotypes in the psychiatric genetics consortium (PGC) are colored purple. Rest of these phenotypes were analyzed using summary statistics obtained from studies based on UK Biobank. The vertical dashed bar corresponds to the genome-wide significance threshold –log_10_(5 × 10^–8^) = 7.3.

Among the cognitive phenotypes analyzed from this resource, none of the phenotypes was associated; a nearly significant association was observed only for intelligence (Table 5). We also analyzed a set of neurological phenotypes and found no association (Table 5). However, we observed significant associations between 5-HTTLPR and human adult height, BMI, total cholesterol, impedance measures to estimate the trunk fat-free mass, and the trunk predicted mass (Fig. 2). Thus, none of 107 psychiatric phenotypes in GWAS Atlas and nine psychiatric phenotypes in PGC was found to be associated with 5-HTTLPR.

**Table 5:** 5-HTTLPR association results obtained by VNTR.s for some cognitive and neurological phenotypes in GWAS atlas (https://atlas.ctglab.nl/).

Phenotype	Phenotypes class	*p* value
Intelligence	Cognitive	5.10E–08
Pairs matching test—number of incorrect matches in round	Cognitive	0.04
Pairs matching test—time to complete round	Cognitive	0.13
Handedness	Cognitive	0.14
Trail making test—duration to complete numeric path (trail #1)	Cognitive	0.16
Symbol digit substitution test—duration to entering value	Cognitive	0.44
Reaction time	Cognitive	0.46
Fluid intelligence test—number of fluid intelligence questions attempted within time limit	Cognitive	0.55
Numeric memory test—maximum digits remembered correctly	Cognitive	0.61
Trail making test—duration to complete alphanumeric path (trail #2)	Cognitive	0.68
Symbol digit substitution test—number of symbol digit matches made correctly	Cognitive	0.82
Symbol digit substitution test—number of symbol digit matches attempted	Cognitive	0.92
Headache	Neurological	0.02
Knee pain	Neurological	0.03
Back pain for 3+ months	Neurological	0.03
Neck or shoulder pain	Neurological	0.06
Hip pain	Neurological	0.3
Migraine	Neurological	0.37
Alzheimer disease	Neurological	0.55
Headaches for 3+ months	Neurological	0.56
Knee pain for 3+ months	Neurological	0.83
Neck/shoulder pain for 3+ months	Neurological	0.88
Stomach or abdominal pain	Neurological	1
Back pain	Neurological	1

## Discussion

We successfully developed and applied a GWAS summary-statistics-based approach to examine the direct effect of the serotonin transporter (SLC6A4) promoter VNTR polymorphism (5-HTTLPR) on susceptibility of neurobehavioral traits and psychiatric disorders in the largest available samples for genetic studies. Despite the long-debated history of this locus as having direct as well as moderating effects on traumatic events and stress on depression [7–10] (and so many other reports of a wide-range of behavioral phenotypes and personality traits that continue to flood the PubMed publication records), we found no evidence of association between the 5-HTTLPR and any of these traits.

We examined a broad range of psychiatric, cognitive, and neurological phenotypes using publicly available summary statistics. The most significant finding was for association between 5-HTTLPR and intelligence [14] but not reaching the well-established genome-wide significance threshold for common allele associations in GWAS studies [15] and without additional correction for multiple testing given the many traits tested in our study. Further analysis of some 400 disorders and traits in the GWAS Atlas belonging to other domain categories (e.g. environment, immunological, metabolic, respiratory, skeletal) and with reasonably powered GWAS sample sizes of *N* > 50,000, of which many are based on the UKBB data [13], yielded no significant results except for adult height [13], total cholesterol [16], BMI [17], and two related traits that were available in the UKBB and included in the GWAS Atlas, i.e., impedance measures to estimate the trunk fat-free mass and the trunk predicted mass. Only adult height, however, remains significant after correction for multiple testing. One of the eight SNPs of the 5-HTTLPR predictor, rs11651241, is strongly associated with adult height, which might explain the strong association signal in our study. By and large, our analysis represents the most systematic effort to examine the role of the serotonin transporter promoter polymorphism 5-HTTLPR in neurobehavioral traits without finding evidence for any direct effect of this VNTR to disease or trait susceptibility. Considering our findings and our understanding of the polygenicity of complex traits, together with the challenge of quantitative phenotyping in the realm of neurobehavioral traits and life events, we conclude that the 5-HTTLPR alleles are also very unlikely to play a measurable role in GxE interactions resulting in human disease and behavior.

Minimac3 is a well-known method for genotype imputation in GWAS [18]. It is based on state space reduction of the hidden Markov model, and is computationally much faster than Minimac2 [19]. Border et al. [20] proposed an efficient approach for imputing genotypes of a VNTR polymorphism using Minimac3 and integrating two reference panel datasets containing genotypes of both VNTR variants and genome-wide SNPs, and the haplotype reference consortium data widely used for imputation [21]. They demonstrated good accuracy of the method and applied it to impute VNTR genotypes in the UKBB. When individual-level genotype data of the SNPs surrounding a VNTR locus are available, this approach is useful for imputing the VNTR genotypes. However, due to multiple steps of analysis involved with the method and complex statistical models underlying Minimac3, it is challenging to design the summary-statistics-based version of the approach. Thus, our method is more useful in the absence of individual-level data.

A limitation of our study is that we considered the biallelic short/long alleles of the VNTR polymorphism instead of considering the actual number of repeats. Even though this is a common strategy for association testing, it does not utilize the complete allelic diversity. Since, our approach is adaptable to the prediction of a multi-allelic VNTR variant, it is possible to extend the method to consider the actual number of repeats in the analysis.

Our approach to using GWAS summary statistics for predicting involvement of a VNTR polymorphism in human complex traits, without the need for individual and large-scale PCR-based genotyping, is not limited to the 5-HTTLPR locus. We applied and developed a computational framework that can easily be expanded to include predictors of other types of genetic and genomic variants for testing their involvement in quantitative and qualitative traits and disorders. We showed that the individual-level genotype data-based method and the summary-statistics-based approach (VNTR.g and VNTR.s) perform equally well, which gives confidence about the reliability of the GWAS summary-statistics-based approach. This method is analogous to that of the transcriptome-wide association studies (TWAS), which we previously developed [12] and successfully applied to identify novel genes associated with complex diseases [22–25]. While the initial TWAS approach is a summary-statistics-based version of predicting gene-expression for association testing, we are now also able to impute (and test for association) the genetic component of allelic variation of more complex genetic and genomic polymorphisms such as VNTRs and other types of recurrent structural variation in the human genome. With the increased efforts of whole genome sequencing of larger cohorts for genetic studies, novel computational methods have been developed to directly test for association of VNTRs, microsatelites, and other repeat structures [26–28]. For now, however, with the extensive data collection and availability of much larger GWAS studies with genome genotyping array data of hundreds of thousands of participants, our approach provides a more efficient way of testing for involvement of VNTRs and other types of haplotypes-associated genetic variation in complex traits.

## Supplementary information

Supplementary material: A summary-statistics-based approach to examine the role of serotonin transporter promoter tandem repeat polymorphism in psychiatric phenotype

## Data Availability

The datasets analyzed during the current study are available from the psychiatric genetics consortium (https://www.med.unc.edu/pgc/), UK Biobank (https://www.ukbiobank.ac.uk/), and GWAS atlas (https://atlas.ctglab.nl/). All data generated during this study are included in this article and its supplementary materials.
