# Supplementary material for: A summary-statistics-based approach to examine the role of serotonin transporter promoter tandem repeat polymorphism in psychiatric phenotypes
Source: Eur J Hum Genet. 2021 Dec 23;30(5):547–54. doi: 10.1038/s41431-021-00996-6 (PMC9091198; doi:10.1038/s41431-021-00996-6)
Supplement: Supplementary file 1 — Supplementary material: A summary-statistics-based approach to examine the role of serotonin transporter promoter tandem repeat polymorphism in psychiatric phenotype [file 41431_2021_996_MOESM1_ESM.pdf]

Supplementary material: A summary-statistics based approach to examine the role of serotonin transporter promoter tandem repeat polymorphism in psychiatric phenotype

Arunabha Majumdar[1*], Preksha Patel[2*], Bogdan Pasaniuc[3,4,5], and Roel A. Ophoff[1,5,6∮]

[1]Department of Mathematics, Indian Institute of Technology Hyderabad, Kandi, Telangana, India

[2]Center for Neurobehavioral Genetics, Semel Institute for Neuroscience and Human Behavior, University of California, Los Angeles, CA, USA

[3]Department of Pathology and Laboratory Medicine, David Geffen School of Medicine, University of California, Los Angeles, CA, USA

[4]Department of Computational Medicine, David Geffen School of Medicine, University of California, Los Angeles, CA, USA

[5]Department of Human Genetics, David Geffen School of Medicine, University of California, Los Angeles, CA, USA

[6]Department of Psychiatry, Erasmus University Medical Center, Rotterdam, The Netherlands

*Joint first author

∮Correspondence: ROphoff@mednet.ucla.edu

Table S1: 5-HTTLPR association p-values obtained by VNTR.s for the second set of psychiatric phenotypes in GWAS atlas (`https://atlas.ctglab.nl/`).

Phenotype	p-value
Happiness	0.32
Recent inability to stop or control worrying	0.35
Reason for reducing amount of alcohol drunk: Illness or ill health	0.36
Spirits intake	0.38
Recent easy annoyance or irritability	0.38
Victim of physically violent crime	0.38
Number of unsuccessful stop-smoking attempts	0.41
Felt very upset when reminded of stressful experince in past month	0.42
Probable major depressive disorder	0.43
Type of tobacco previously smoked: Manufactured cigarettes	0.43
Impact of normal roles during worst period of depression	0.46
Past tobacco smoking	0.48
Frequency of depressed mood in last 2 weeks	0.48
Bipolar/Major depression	0.49
Smoking/smokers in household	0.49
Tobacco smoking	0.50
Ever suffered mental distress preventing usual activities	0.50
Excessive daytime sleepiness	0.51
Red wine intake	0.51
Repeated disturbing thoughts of stressful experience in past month	0.54
Ever felt worried, tense, or anxious for most of a month or longer	0.54
Frequency of tenseness / restlessness in last 2 weeks	0.55
Recent trouble concentrating on things	0.56
Did your sleep change?	0.56
Feelings of tiredness during worst episode of depression	0.57
Recent poor appetite or over eating	0.57
Diagnosed with life-threatening illness	0.58
Recent changes in speed/amount of moving or speaking	0.59
Age at first episode of depression	0.59
Recent feelings of depression	0.60
Happiness with own health	0.60
Why stopped smoking: Illness or ill health	0.62
Feelings of worthlessness during worst period of depression	0.63
Able to confide	0.65
Seen doctor (GP) for nerves, anxiety, tension or depression	0.66
Ever had known person concerned about, or recommended reduction of alcohol consumption	0.66
Ever highly irritable/argumentative for 2 days	0.66
Recent feelings of foreboding	0.69

Table S2: 5-HTTLPR association p-values obtained by VNTR.s for the third set of psychiatric phenotypes in GWAS atlas.

Phenotype	p-value
Ever sought or received professional help for mental distress	0.70
Beer/cider intake	0.70
Recent feelings of inadequacy	0.71
Ever had prolonged loss of interest in normal activities	0.75
Why stopped smoking: Health precaution	0.75
Nicotine dependence	0.76
Recent feelings or nervousness or anxiety	0.77
Avoided activities or situations because of previous stressful experience in past month	0.77
Seen a psychiatrist for nerves, anxiety, tension or depression	0.81
Felt irritable or had angry outbursts in past month	0.81
Belief that own life is meaningful	0.81
Duration of worst depression	0.82
Age at last episode of depression	0.84
Ever taken cannabis	0.85
Age stopped smoking	0.86
Cigarettes per day	0.86
Age of smoking initiation	0.87
Alcohol usually taken with meals	0.88
Ever stopped smoking for 6+ months	0.88
Risk taking	0.88
Recent feelings of tiredness or low energy	0.88
Depression	0.89
Recent lack of interest of pleasure in doing things	0.90
Alcohol intake	0.91
Ever unenthusiastic/disinterested for a whole week	0.92
Recent thoughts of suicide or self-harm	0.92
Witnessed sudden violent death	0.92
Professional informed about depression	0.93
Felt distant from other people in past month	0.93
Difficulty concentrating during worst depression	0.96
Alcohol intake versus 10 years previously	0.98

Table S3: Coefficients of the prediction model to impute 5-HTTLPR genotypes fitted by Lu et al. (2012). The order of the tag SNPs is denoted by $i = 1, \ldots, 8$. The coefficients consist of two vectors $a_1 = (a_{11}, a_{21}, \ldots, a_{81})$ and $a_2 = (a_{21}, a_{22}, \ldots, a_{82})$ and two intercept terms b_1, b_2.

SNP	i	a_{i1}	a_{i2}
rs7217677	1	-0.053427738	-0.017944884
rs4494608	2	0.053488163	0.010513604
rs1487971	3	-0.388960505	-0.081862677
rs11651241	4	-0.212066944	-0.0539594
rs2129785	5	-0.229855845	-0.048378268
rs887469	6	-0.073285608	-0.024940628
rs4794873	7	0.111159158	0.034747773
rs1061342	8	-0.059042871	-0.021787721
–		b_1	b_2
–		-0.048664235	-0.201663866

*For more details about the prediction model, see the paper by Lu et al. (A Lu, S Bakker, E Janson, S Cichon, R Cantor, and R Ophoff, Prediction of serotonin transporter promoter polymorphism genotypes from single nucleotide polymorphism arrays using machine learning methods. Psychiatric genetics, 22(4):182 - 188, 2012).

Table S4: GWAS p-values of marginal association between each pair of a tag SNP of 5-HTTLPR and a psychiatric phenotype in the psychiatric genetics consortium (`https://www.med.unc.edu/pgc/`).

	rs7217677	rs4494608	rs1487971	rs11651241	rs2129785	rs887469	rs4794873	rs1061342
Bipolar disorder	0.12	0.83	0.02	0.53	0.44	0.29	0.02	0.06
Attention deficit hyperactivity disorder	0.15	0.61	0.03	0.22	0.09	0.06	0.35	0.33
Eating disorder	0.001	0.22	0.52	0.79	0.004	0.44	0.95	0.65
Post traumatic stress disorder	0.75	0.22	0.32	0.37	0.93	0.14	0.07	0.16
Schizophrenia	0.04	0.24	0.44	0.25	0.53	0.59	0.16	0.005
Autism spectrum disorder	0.72	0.16	0.99	0.35	0.48	0.37	0.56	0.26
Major depressive disorder	0.005	0.53	0.52	0.96	0.75	0.39	0.04	0.02
Tourette syndrome	0.10	0.29	0.31	0.10	0.31	0.53	0.78	0.81
Alcohol dependence	0.33	0.35	0.69	0.64	0.88	0.29	0.08	0.68
